# Synaptic Alterations in a Transgenic Model of Tuberous Sclerosis Complex: Relevance to Autism Spectrum Disorders

**DOI:** 10.3390/ijms221810058

**Published:** 2021-09-17

**Authors:** Grzegorz A. Czapski, Lidia Babiec, Henryk Jęśko, Magdalena Gąssowska-Dobrowolska, Magdalena Cieślik, Marta Matuszewska, Małgorzata Frontczak-Baniewicz, Karolina Zajdel, Agata Adamczyk

**Affiliations:** 1 Department of Cellular Signalling, Mossakowski Medical Research Institute, Polish Academy of Sciences, Pawińskiego 5, 02-106 Warsaw, Poland; gczapski@imdik.pan.pl (G.A.C.); lbabiec@imdik.pan.pl (L.B.); hjesko@imdik.pan.pl (H.J.); mgassowska@imdik.pan.pl (M.G.-D.); mcieslik@imdik.pan.pl (M.C.); mmatuszewska@imdik.pan.pl (M.M.); 2 Electron Microscopy Platform, Mossakowski Medical Research Institute, Polish Academy of Sciences, Pawińskiego 5, 02-106 Warsaw, Poland; mbaniewicz@imdik.pan.pl (M.F.-B.); kzajdel@imdik.pan.pl (K.Z.)

**Keywords:** tuberous sclerosis complex, synaptic dysfunction, autism, animal model

## Abstract

Tuberous sclerosis complex (TSC) is a rare, multi-system genetic disease with serious neurological and mental symptoms, including autism. Mutations in the *TSC1*/*TSC2* genes lead to the overactivation of mTOR signalling, which is also linked to nonsyndromic autism. Our aim was to analyse synaptic pathology in a transgenic model of TSC: two-month-old male B6;129S4-Tsc2^tm1Djk/J^ mice with Tsc2 haploinsufficiency. Significant brain-region-dependent alterations in the expression of several synaptic proteins were identified. The most prominent changes were observed in the immunoreactivity of presynaptic VAMP1/2 (ca. 50% increase) and phospho-synapsin-1 (Ser62/67) (ca. 80% increase). Transmission electron microscopy demonstrated serious ultrastructural abnormalities in synapses such as a blurred structure of synaptic density and a significantly increased number of synaptic vesicles. The impairment of synaptic mitochondrial ultrastructure was represented by excessive elongation, swelling, and blurred crista contours. Polyribosomes in the cytoplasm and swollen Golgi apparatus suggest possible impairment of protein metabolism. Moreover, the delamination of myelin and the presence of vacuolar structures in the cell nucleus were observed. We also report that Tsc2^+/−^ mice displayed increased brain weights and sizes. The behavioural analysis demonstrated the impairment of memory function, as established in the novel object recognition test. To summarise, our data indicate serious synaptic impairment in the brains of male Tsc2^+/−^ mice.

## 1. Introduction

Tuberous sclerosis complex (TSC) is an autosomal, dominant genetic disease evoked by the mutation of either the *TSC1* or *TSC2* gene; these genes encode the proteins Tsc1 (hamartin) and Tsc2 (tuberin), respectively. The disorder affects approximately 1 in 5800 people and is typically characterised by widespread hamartomas in multiple organ systems, including the brain [1]. In the brain, hamartin and tuberin have been implicated in the control of cortical lamination, the regulation of dendritic arborization, neuronal migration, polarization, axonal outgrowth, and spine formation [2,3]. Pathological mutations in these genes disrupt the interaction between hamartin and tuberin and affect cell function via the improper regulation of the mechanistic/mammalian target of rapamycin (mTOR) signalling cascade and multiple downstream effectors responsible for the modulation of key cellular processes, including protein synthesis, autophagy, cell growth, and proliferation [4]. Specifically, in the brain, the mTOR signalling pathway is involved in the regulation of synaptogenesis, corticogenesis, the connectivity of neuronal circuits, the proliferation of cerebral cells, and dendritic spine density [5]. In particular, the hamartin-tuberin complex inhibits the activity of Ras-family GTPase, the small G-protein Rheb (Ras homolog enriched in the brain), which plays a critical role in the activation of mTOR kinase. Therefore, the dysfunction of hamartin and/or tuberin leads to the overactivation of the mTOR kinase-dependent pathway. The serine/threonine protein kinase mTOR exists in two diverse complexes: mTORC1 (mTOR, Raptor, and LST8) and mTORC2 (mTOR, Rictor, LST8, and SIN1). Upon stimulation by growth factors, signals related to nutrient availability, or hypoxia, mTOR phosphorylates a variety of substrates, affecting numerous essential cell processes, including the regulation of several components involved in protein synthesis, such as initiation and elongation factors, and the biogenesis of ribosomes themselves. mTOR-dependent translation is regulated by the control of two critical core components of the translation-initiation machinery: ribosomal protein S6 kinase beta-1 (S6K1 or p70S6K) and the eIF-4E-binding protein 1 (4E-BP1). S6K1 is phosphorylated by mTOR at Ser389. After activation, S6K1 phosphorylates the ribosomal subunit protein S6, leading to an increase in protein translation. 4E-BP1 is a repressor of the translation-initiation complex (eIF-4E), which is required for the 5′-cap-dependent translation of mRNA. When phosphorylated by mTOR, 4E-BP1 dissociates from eIF-4E, allowing for the initiation of 5′-cap-dependent RNA translation. A growing body of evidence indicates that the mTOR pathway is the central regulator of cap-dependent local translation at the synapse [6]. Disturbances in local-translation control and changes in the quantities of proteins at synapses could affect synaptic structure and function and might be responsible for a shift in the balance of synaptic plasticity and associated processes of learning and memory as well as behaviour. Therefore, the dysregulation of synaptic protein synthesis at synapses has been implicated in the pathogenesis of several diseases, including autism, epilepsy, and neurodegenerative disorders [7,8].

In addition, the overactivation of the mTOR pathway leads to the impairment of autophagy [9]. Overactivated mTORC1 phosphorylates ULK1 (Unc-51-like autophagy-activating kinase) at Ser757, leading to its inhibition and, consequently, a decrease in autophagic activity [10,11]. However, in *Tsc2*-knockdown neurons, the accumulation of the autophagic marker LC3-II and of the autophagy substrate p62 has been observed [9]. The data suggest that, in neurons, despite the mTOR-dependent inhibitory phosphorylation of ULK1 at Ser757, increased AMPK-dependent activating phosphorylation of ULK1 at Ser555 occurs, leading, in effect, to increased autophagic activity through the AMPK-dependent activation of ULK1.

Nevertheless, abnormalities in the mTOR-dependent signalling pathway have been suggested in the pathology of several disorders, from neurodevelopmental syndromes in which autism is highly prevalent, to neurodegenerative disorders such as Alzheimer’s disease (AD) and Parkinson’s disease (PD) [12,13,14]. There is convincing evidence that mutations in the *TSC2* gene evoke more severe neurological manifestations, including intellectual disability, than mutations in the *TSC1* gene [15,16,17,18].

Therefore, the aim of our study was to determine the effect of haploinsufficiency of *Tsc2* on the expression of synaptic proteins responsible for the regulation of neurotransmitter release and neuronal plasticity. Structural changes in synapses were analysed using transmission electron microscopy (TEM), and appropriate behavioural tests were performed. We identified alterations in the expression of key synaptic proteins and serious ultrastructural changes at synapses in the brains of male Tsc2^+/−^ mice, as compared to Tsc2^+/+^ animals. Moreover, memory function was impaired, as demonstrated in the novel object recognition test; however, exploratory and social behaviours were not affected in Tsc2^+/−^ mice.

## 2. Results

**Figure 1:**
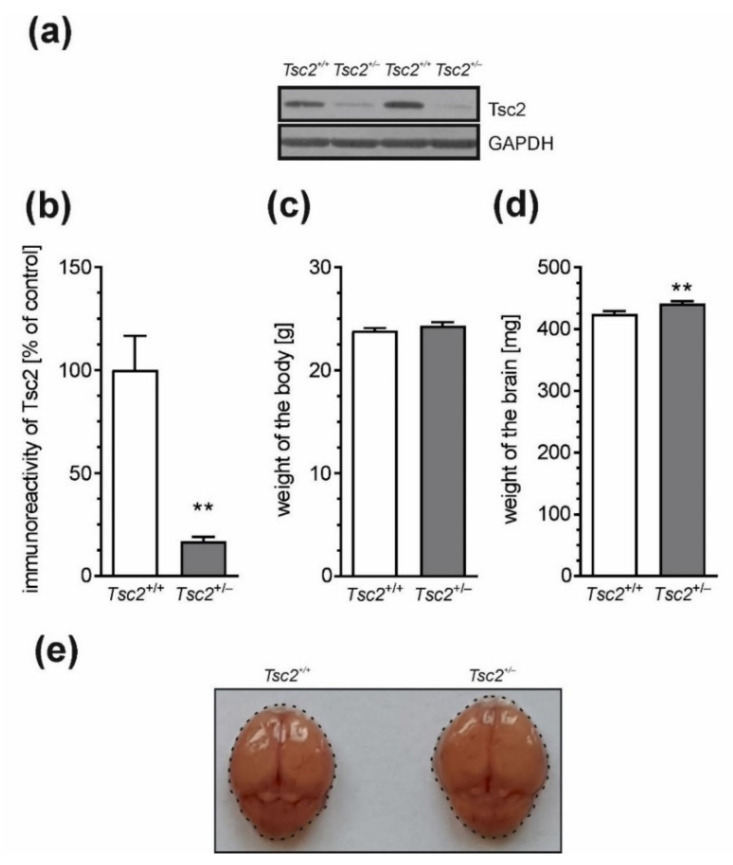
The effect of Tsc2 haploinsufficiency on body weight in mice. (**a**) The protein level of Tsc2 in the brain cortex of Tsc2^+/+^ and Tsc2^+/−^ two-month-old male mice was analysed by Western blotting. Typical blots are presented. (**b**) Densitometric analysis of immunoreactivity of Tsc2 in the brain cortex. Data were normalised to GAPDH. *n* = 3. (**c**,**d**) The effect of Tsc2 insufficiency on the body weight and brain weight. (**e**) Example photographs of the brains of Tsc2^+/+^ and Tsc2^+/−^ two-month-old male mice. *n* = 30. Data represent the mean values ± SEMs. ** *p* < 0.01 vs. wild-type animals, as determined by Student’s *t*-test.

In our study, we used strain B6;129S4-Tsc2^tm1Djk/J^, which is a murine model of TSC. As shown in Figure 1a,b, the level of the Tsc2 protein was significantly reduced in the TSC murine model compared to the control wild-type (wt) Tsc2^+/+^ animals. Consequently, processes downstream of Tsc2 and the mTOR pathway were affected, as demonstrated by alterations of the brain weights in Tsc2^+/−^ mice, suggesting impaired development of the central nervous system and megalencephaly. The average weight of the animal’s brain was increased by 3.42 percent in Tsc2^+/−^ mice, whereas the weight of the body was not changed (Figure 1c–e).

To reveal the impact of Tsc2 haploinsufficiency on the expression of synaptic neurotransmission-related proteins, the mRNA and protein levels were analysed by using qPCR and Western blotting, respectively. Calcium-binding synaptic vesicle protein synaptotagmin-1 (Syt1); phosphorylated synapsin (p-Syn), which is responsible for synaptic vesicle docking, fusion, and recycling; the SNARE proteins VAMP1/2, syntaxin 1 (Stx1), and SNAP25; and neuroligins (Nlgn), cell-adhesion molecules that maintain trans-synaptic connections, were investigated in the cerebral cortex, hippocampus (regions CA1/CA2 and CA2/CA3), and cerebellum. It was observed that the expression of specific synaptic proteins was significantly changed in Tsc2^+/−^ mice (Table 1). The mRNA levels for *Vamp1*, *Vamp2*, *Syt1* and *Nlgn1* were modestly increased in the brain cortex of Tsc2^+/−^ mice, whereas the expression of these genes in the hippocampus and cerebellum was not changed. The expression of *Stx1a*, *Stx1b*, *Snap25* and *Nlgn3* was not altered in any of the studied brain structures.

**Figure 2:**
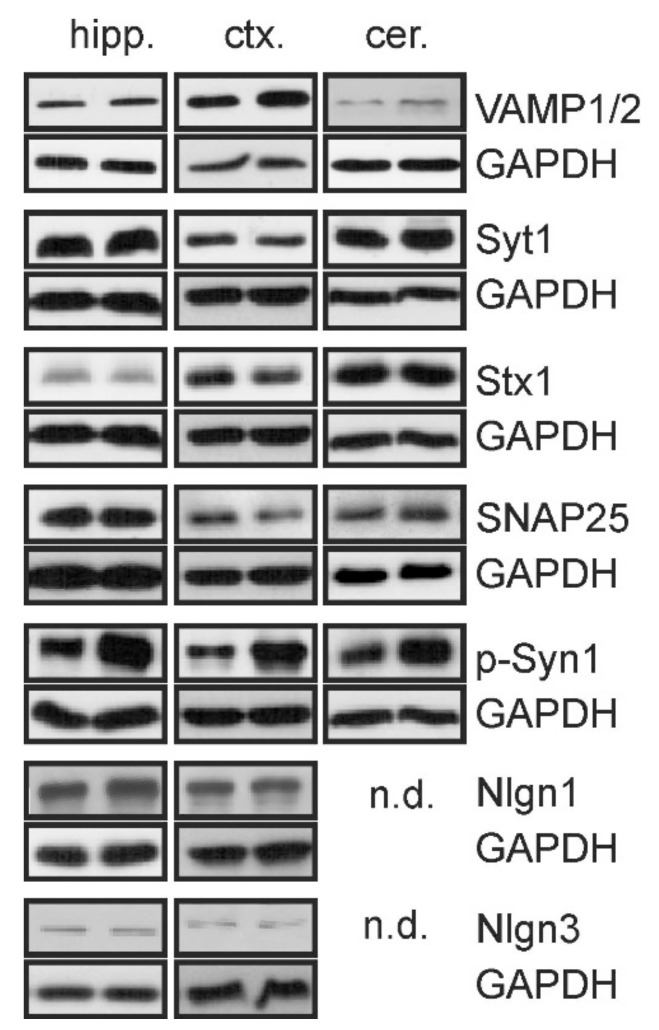
The effect of Tsc2 haploinsufficiency on levels of synaptic proteins. The immunoreactivity of synaptic proteins in the hippocampus (hipp.), cortex (ctx.), and cerebellum (cer.) was determined by using Western blotting. Representative pictures are presented. GAPDH was used as a loading control. n.d.—not detected; densitometric analysis is presented in Table 2.

Concomitantly, significant changes in the levels of key synaptic proteins were observed (Figure 2, Table 2). The level of VAMP1/2 was significantly (*p* < 0.05) increased in the hippocampus, cerebral cortex, and cerebellum, whereas the levels of Stx1 and Syt1 were reduced in the cerebral cortex but were unchanged in the hippocampus and cerebellum. Interestingly, the immunoreactivity of phospho-synapsin (Ser62/67) was significantly (*p* < 0.01) increased in the hippocampus and cerebral cortex. The levels of the other tested synaptic proteins were not changed in any of the analysed brain structures. Immunoreactivity of neuroligins 1 and 3 was not detected in the cerebellum.

Since the amount and availability of synaptic proteins affect not only synaptic signalling but also the structure and plasticity of neurons, and the fact that changes in synaptic morphology and number are linked to the majority of psychiatric and neurological disorders, from autism to neurodegenerative disorders, a comprehensive ultrastructural analysis of the brains of Tsc2^+/−^ mice using transmission electron microscopy (TEM) was performed.

**Figure 3:**
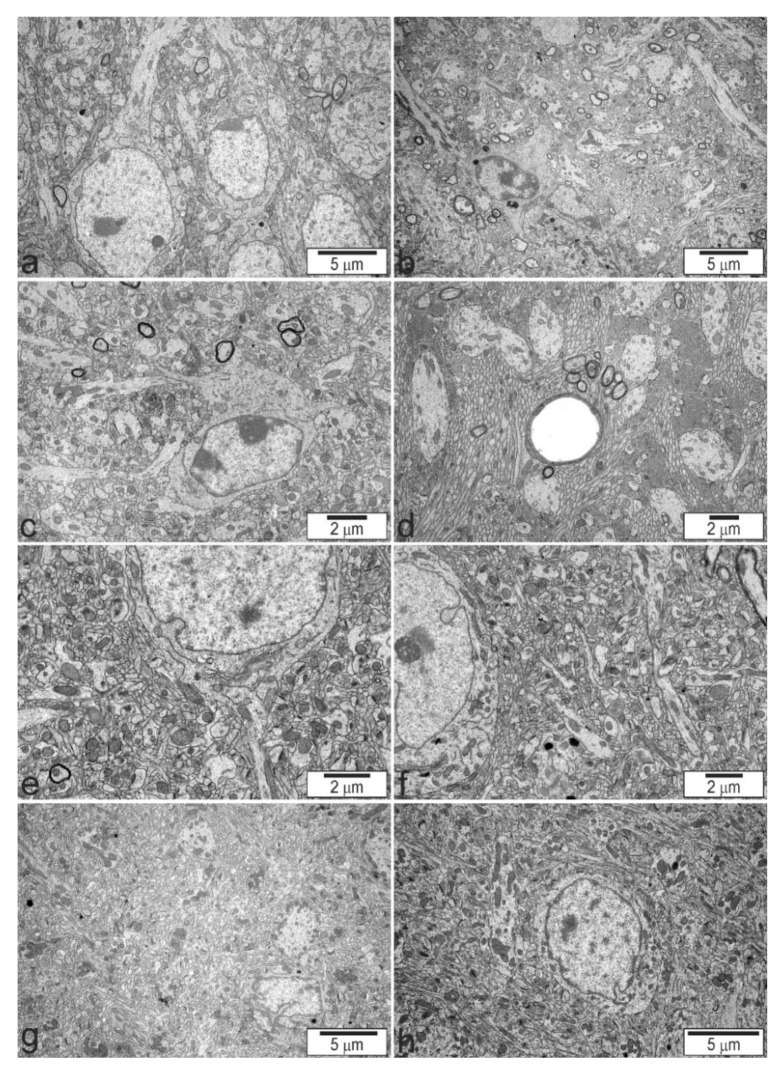
The effect of Tsc2 haploinsufficiency on the ultrastructure of neuronal cells in the CA1/CA2 (**a**,**b**). and CA2/CA3 (**c**,**d**) regions of the hippocampus, brain cortex (**e**,**f**), and cerebellum (**g**,**h**). (**a**,**c**,**e**,**g**)—control group Tsc2^+/+^; (**b**,**d**,**f**,**h**)—Tsc2^+/−^ group. Typical electronograms at low magnification are presented.

**Figure 4:**
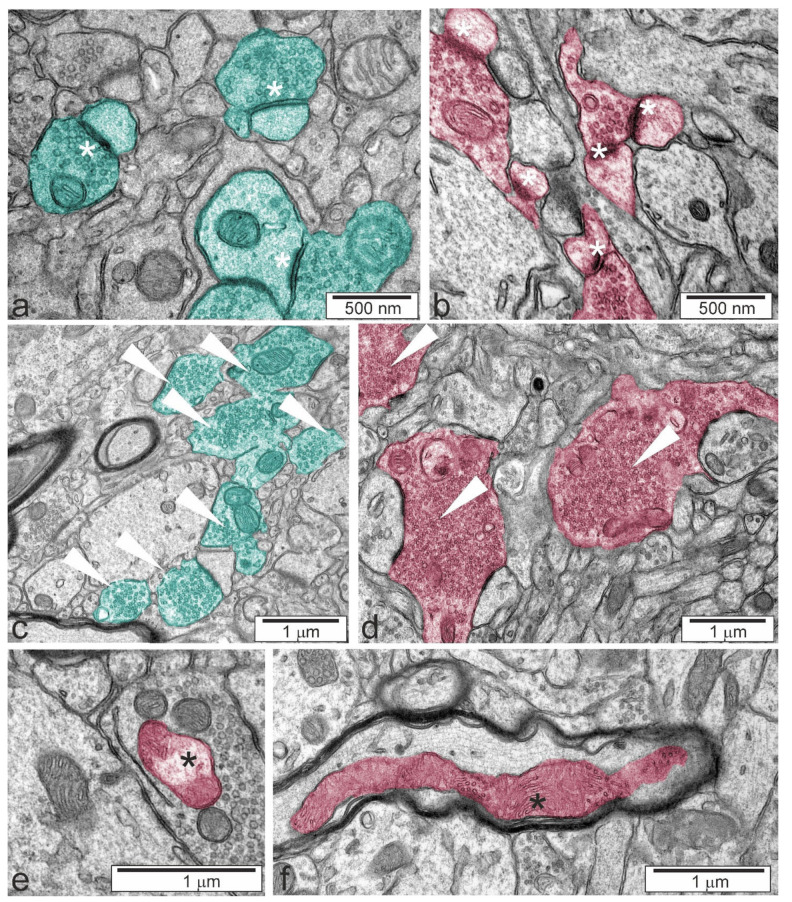
Typical electronograms presenting morphological alterations of neuronal cells in the hippocampus. (**a**) Correct synaptic density (white asterisk), (**b**) blurred structure for synaptic density (white asterisk), (**c**) correct synapses (arrowheads), (**d**) overloaded synaptic endings (arrowheads), (**e**) swollen mitochondrium (black asterisk), and (**f**) excessively elongated mitochondrium (black asterisk). The colour was used to label control (turquoise) and altered (reddish) structures.

**Figure 5:**
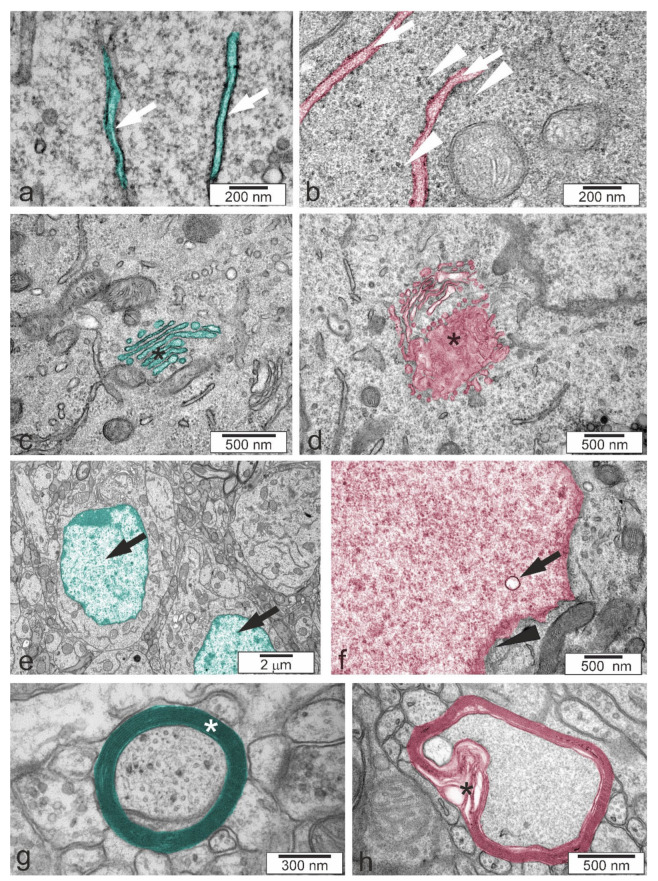
Typical electronograms presenting morphological alterations of neuronal cells in the hippocampus. (**a**) Correct cytoplasm and endoplasmic reticulum (white arrow), (**b**) swollen endoplasmic reticulum (white arrows) and polyribosomes in the cytoplasm (white arrowheads), (**c**) correct Golgi apparatus (black asterisk), (**d**) swollen Golgi apparatus (black asterisk), (**e**) correct nuclei (black arrows), (**f**) vacuolar structures in the cell nucleus (black arrow) and diffused nuclear envelope (black arrowhead), (**g**) correct structure of myelin (white asterisk), and (**h**) delamination of myelin (black asterisk). The colour was used to label control (turquoise) and altered (reddish) structures.

**Figure 6:**
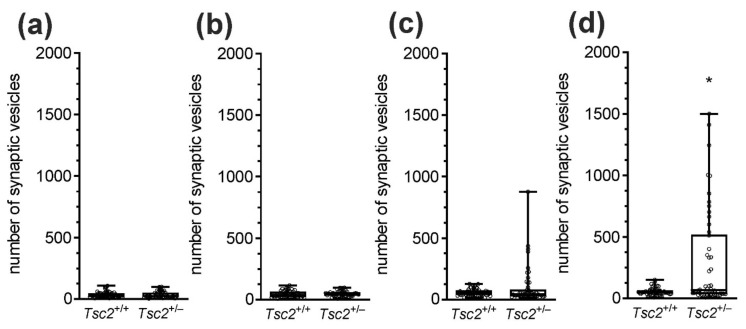
The effect of Tsc2 haploinsufficiency on the number of synaptic vesicles in (**a**) cerebral cortex, (**b**) cerebellum, (**c**) CA1/CA2 region of the hippocampus, and (**d**) CA2/CA3 region of the hippocampus. The quantitative analysis of microphotographs was performed for 5 animals in each group; 10 random synapses from each animal were taken for analysis (*n* = 50). Data were not normally distributed and are presented as medians with interquartile ranges, minimums, and maximums. * *p* ˂ 0.05 vs. wild-type animals, as determined using the Mann–Whitney test.

We analysed neurons in the cerebral cortex, cerebellum, and two regions of the hippocampus, CA1/CA2 and CA2/CA3. A full list of the pathological alterations in all the analysed brain structures of Tsc2^+/−^ mice is presented in Table 3. Example microphotographs showing control tissue from Tsc2^+/+^ animals and typical alterations in Tsc2^+/−^ animals are presented in Figure 3, Figure 4 and Figure 5. The analysis demonstrated that Tsc2 haploinsufficiency evoked prominent pathological changes in the morphology of neurons. We observed a blurred structure of synaptic density (Figure 4a,b) and a significantly increased number of synaptic vesicles (Figure 4c,d). Some synapses were extremely overloaded with synaptic vesicles in the brains of Tsc2^+/−^ animals. Numerous overloaded synaptic endings were observed exclusively in the hippocampus; very few were observed in the cortex and cerebellum. Semi-quantitative analysis of the average number of synaptic vesicles was performed. As demonstrated in Figure 6, a statistically significant difference in the number of synaptic vesicles in the synaptic ending was confirmed only in the CA2/CA3 region of the hippocampus. We also detected impairment of mitochondrial ultrastructure, represented by swelling and blurred crista contours. The mitochondrial pathology was typically observed in synaptic endings. Additionally, significantly elongated mitochondria with blurred constrictions were observed, which was distinctively different from those in the control tissue from Tsc2^+/+^ animals, where the mitochondria were predominantly of ball-like structure or sausage-shaped (Figure 4e,f). These morphological differences may be due to the different patterns in the expression of fission/fusion proteins as well as defects in mitophagy since mTOR kinase is involved in the regulation of both phenomena [19]. We found that some of the endoplasmic reticulum networks were fragmented, with polyribosomes freed in the cytosol (Figure 5a,b). Moreover, the swelling of the Golgi apparatus in Tsc2^+/−^ animals was evident (Figure 5c,d). Untypical vacuolar structures were observed in the neuronal nuclei, which may indicate certain carioskeleton disorders (Figure 5e,f). Additionally, we observed the delamination of myelin in Tsc2^+/−^ mice (Figure 5g,h).

**Figure 7:**
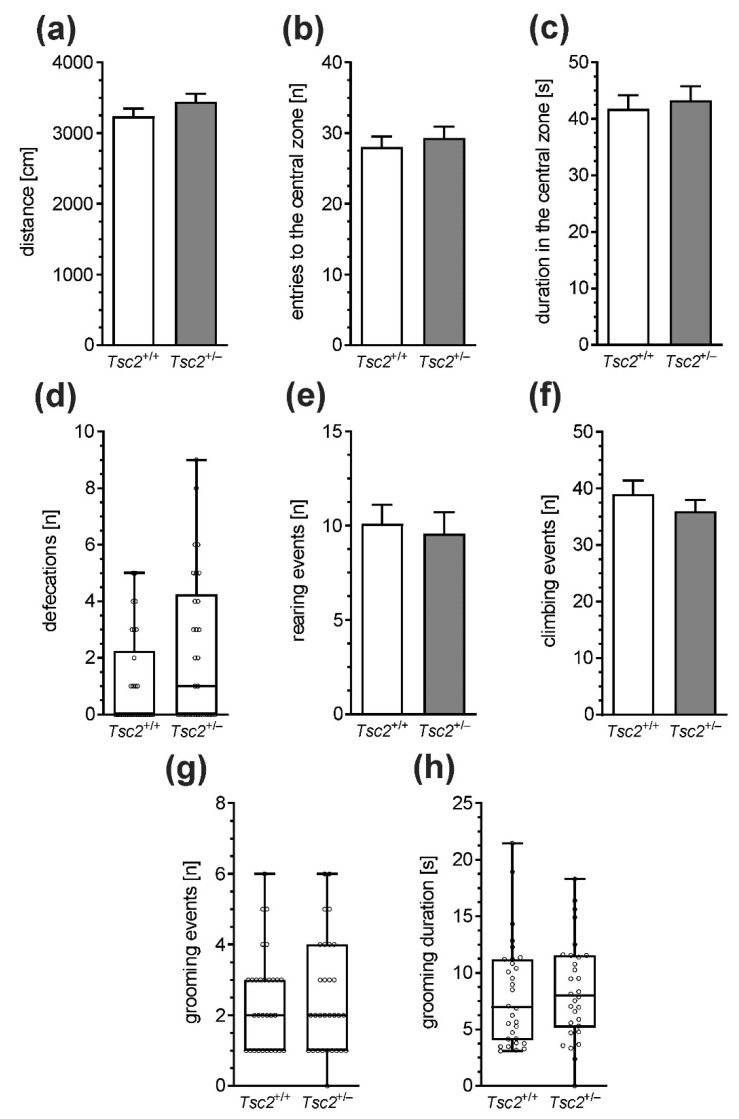
The effect of Tsc2 haploinsufficiency in mice on behaviour (exploratory activity). The exploratory activity of male mice was analysed in an open-field test. (**a**) The total distance travelled by animals, (**b**) the number of entries to the central zone, (**c**) the time spent in the central zone, (**d**) the number of defecation events, (**e**) the number of rearing events, (**f**) the number of climbing events, (**g**) the number of grooming events, and (**h**) the total time spent on self-grooming. Data (**a**–**c**,**e**,**f**) represent the mean values ± SEMs from *n* = 30 independent experiments. Data (**d**,**g**,**h**) not normally distributed are presented as medians with interquartile ranges, minimums, and maximums (*n* = 30). Each data point is from a separate animal.

**Figure 8:**
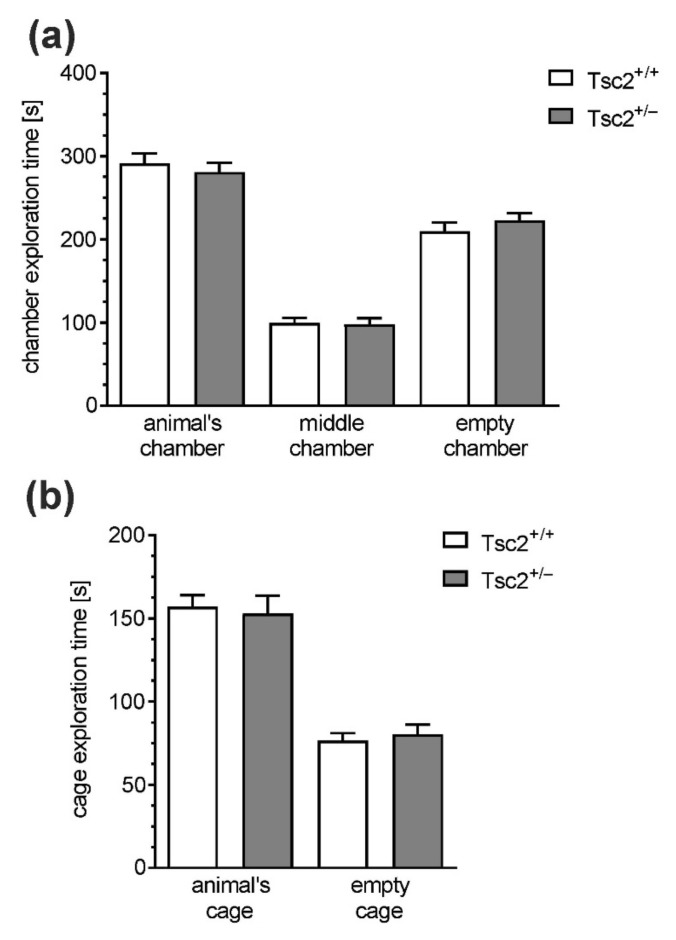
The effect of Tsc2 haploinsufficiency in mice on behaviour (sociability). (**a**) The time spent by the tested animal in the chamber; (**b**) the time spent by the tested animal on the exploration of cages. Data represent the mean values ± SEMs from *n* = 20 independent experiments. Each data point is from a separate animal.

**Figure 9:**
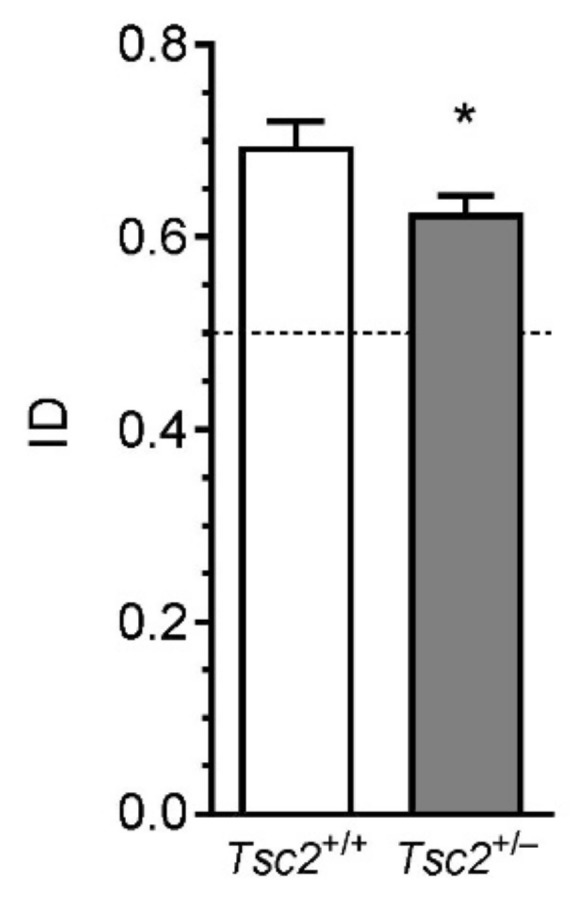
The effect of Tsc2 haploinsufficiency in mice on behaviour (cognitive function). The memory of male mice was analysed in a novel object recognition test. Index of discrimination (ID) was calculated, as described in the Methods section. Presented results are means ± SEMs from *n* = 10 animals in each group. * *p* < 0.05, compared to control, as determined using Student’s *t*-test. Each data point is from a separate animal.

To examine whether Tsc2^+/−^ haploinsufficiency was associated with behavioural deficits, several aspects of animals’ behaviour were analysed. As shown in Figure 7, exploratory activity and anxiety-related behaviours were not changed in Tsc2^+/−^ mice, as determined in the open-field test. The distance travelled during the test (Figure 7a) and the exploration of the central zone (Figure 7b,c) were the same in Tsc2^+/+^ and Tsc2^+/−^ animals. Although the numbers of episodes of defecation (Figure 7d), rearings (Figure 7e), climbing (Figure 7f), and grooming (Figure 7g,h) behaviour did not show statistically significant differences between Tsc2^+/+^ and Tsc2^+/−^ animals (*p* > 0.05), a weak but tenacious drift toward increased anxiety in Tsc2^+/−^ mice was visible. Because TSC haploinsufficiency is known to induce autistic behaviours in patients, a three-chamber Crowley’s test was also performed. In this test, the animal is allowed to freely investigate two chambers connected by a third one. The first chamber contains a cage with another animal, and the second chamber contains an empty cage. Wild-type animals prefer staying in the chamber with another animal. When social behaviour is impaired, the preference toward an animal’s cage is significantly reduced. As shown in Figure 8a, both Tsc2^+/+^ and Tsc2^+/−^ mice spend significantly more time on the exploration of the chamber with the unknown animal than on the exploration of the chamber containing an empty cage; however, no difference between Tsc2^+/+^ and Tsc2^+/−^ was observed. The analysis of the time spent on the direct investigation of the cages provides more specific results. As shown in Figure 8b, the cage with an animal was investigated for significantly longer by the tested animal than the empty cage; however, no differences between Tsc2^+/+^ and Tsc2^+/−^ were detected. Finally, we performed a novel object recognition test. As shown in Figure 9, the index of discrimination (ID) in Tsc2^+/−^ mice is reduced compared to that in Tsc2^+/+^ mice.

## 3. Discussion

In our study, we used a commercially available murine transgenic model of TSC. In the strain B6;129S4-Tsc2^tm1Djk/J^, the gene *Tsc2* was inactivated by the insertion of the *neo* cassette into exon 2 [20]. Tsc2^−/−^ homozygosity is lethal in the embryonic stage. Tsc2^+/−^ mice with Tsc2 immunoinsufficiency develop symptoms resembling human TSC. Because of the deficiency of Tsc2, the activity of the Tsc1/Tsc2 complex is decreased, leading to the overactivation of the mTOR pathway, which is the primary cause of TSC-related symptoms, including tumours of the brain, skin, heart, lungs, and kidney [1,21]. In the brain, cortical tubers are typical abnormalities [22]. The presence of tuber-like lesions has been reported in several rodent models of TSC [23,24,25]. However, in other experimental models, despite some typical TSC symptoms being mimicked, the presence of cortical tubers has not been observed, suggesting that the neurocognitive symptoms may, rather, be caused by the impairment of neuronal connectivity or signalling [26,27]. These pathological tuber-like alterations were also not observed in our study in two-month-old animals.

TSC patients have many neurological and mental symptoms, including autism [1]. A growing body of evidence indicates that the neurobiological basis of autism in TSC may be abnormal synaptic plasticity and alterations in neural connectivity [28,29]. Hamartin (Tsc1) and tuberin (Tsc2) regulate synaptic growth and function in neurons, and their loss causes enlarged spines [30]. A hyperactive mTOR pathway, as a result of Tsc2 insufficiency, is probably responsible for the autism-related symptoms in TSC. The mTOR pathway plays essential roles in the translational machinery, protein-synthesis-dependent long-term potentiation (LTP), synaptic plasticity, and neural network and connectivity formation. The mTOR-kinase-dependent regulation of the activity of the mRNA cap-binding factor eIF4E and S6K is a major effector mechanism through which synaptic protein synthesis is modulated [31]. Kelleher and Bear hypothesised that an increase in the activity of the mTOR pathway could lead to excessive plasticity-related synaptic protein synthesis that may result in changes in synaptic connectivity and cognitive impairment and be one of several pathogenic mechanisms leading to autism [31]. The impairment of synaptic plasticity was also reported in a rat model of TSC (Tsc2^+/−^ rats) [32].

In the current research, we identified alterations in synaptic proteostasis and synaptic ultrastructure impairments, as we previously observed in animal models of autism evoked by environmental factors [33,34]. Concomitantly, a higher synaptic protein level was observed in fragile X syndrome (FXS), a single-gene disorder associated with autism [31]. In the present study, we showed a significantly higher level of key presynaptic proteins involved in the kinetics of synaptic vesicles (SVs), fusion, and neuroexocytosis, such as p-synapsin and synaptobrevin (VAMP1/2). Both p-synapsin and VAMP1/2 were increased in the cerebral cortex and hippocampus; however, in the cerebellum, only VAMP1/2 was upregulated. Changes in synaptic protein expression are dependent on the brain structure, and the greatest disturbance of synaptic protein homeostasis has been observed in the cerebral cortex, where, additionally, a deficit of synaptotagmin and syntaxin-1 has been demonstrated.

Synapsins are a family of phosphoproteins that are the most abundant on SVs. They are involved in the regulation of various synaptic functions such as the formation of presynaptic terminals, the regulation of the vesicle reserve pool at presynaptic terminals, synaptogenesis, and synaptic vesicle docking. Synapsin-1, the major isoform in neurons, plays a key role in the regulation of the SV life cycle and maintains a reserve pool of SVs in the vicinity of the active zone. The binding of synapsin-1 to SV depends on the degree of its phosphorylation. In the dephosphorylated form, synapsin-1 binds to SV, whereas the phosphorylated form dissociates from SVs and enables their translocation to the plasma membrane, their entry to the path of exocytosis, and the release of neurotransmitters. Since synapsin-1 in its phosphorylated form plays a key role in mediating the trafficking of SVs to the active zone, an increase in its level could be responsible for increased neurotransmitter release. Interestingly, this neuron-specific phosphoprotein is expressed with divergent contents and plays diverse roles in excitatory glutamatergic and inhibitory GABAergic neurons [35,36]. Synapsins maintain the reserve pool of glutamatergic vesicles, while regulating the size of the readily releasable pool of GABAergic vesicles. Therefore, we suggest that the mTOR-dependent upregulation of p-synapsin may underlie the E/I imbalance observed in autism spectrum disorders (ASD). Like synapsin-1, VAMP1/2 is localised to SVs and plays an important role in the regulation of neuroexocytosis and the kinetics of neurotransmitter release. Together with syntaxin-1 and SNAP-25, VAMP1/2 forms the SNARE complex that provides the energy to drive fusion. Changes in the expression and activity of any of the SNARE proteins could affect SV fusion, neurotransmitter release, and plasticity. Higher expression of VAMP1/2 could promote SNARE complex formation and exocytosis. In our study, we observed a significant increase in both the mRNA and protein levels of VAMP1/2 in the cerebral cortex and higher protein levels in the hippocampus and cerebellum. Changes in VAMP1/2 levels were also observed in the experimental models of ASD. Prenatal exposure to polyinosinic–polycytidylic acid (PIC) evoked an increase in VAMP1/2 levels in offspring [37]. Similarly, prenatal exposure to valproic acid (VPA) induced an increase in the VAMP1/2 level in the hippocampus but not in the cerebral cortex [33]. In the present work, despite the increase in the level of VAMP1/2, we observed a significantly lower level of syntaxin-1 in the cerebral cortex, which can lead to the dysfunction of the SNARE complex and impaired neurotransmission. Syntaxin-1 is a membrane protein localised to the plasma membrane of the presynaptic active zone and involved in vesicle fusion. In addition to participating in SNARE complex formation, syntaxin-1 binds synaptotagmin on SVs, allowing SV fusion with the plasma membrane. The level of the latter protein is also lowered in the cerebral cortex, despite increased expression of the *Syt1* gene, indicating disturbances of SV cycling in this brain structure. We also noted a higher expression of neuroligin-1 in the cerebral cortex, which, however, is not reflected in the protein level. Therefore, this phenomenon has no biological effect. All these results indicate that specific presynaptic molecules involved in the SV recycling events, the regulation of synaptic plasticity, and brain development are affected by mTOR-kinase-dependent signalling. The activation of this molecular machinery regulating synaptic protein synthesis could be responsible for pathological changes in synaptic structure, function, and plasticity. mTOR is also a key regulator of gene transcription, so its overactivation significantly affects the activity of several transcription factors (e.g., STAT3, PPAR-γ, HIF-1α, TFEB, and YY1) and the expression of many genes [38]. Moreover, the mTOR pathway also regulates epigenetic mechanisms [39]. A recent study demonstrated a general decrease in the hippocampal histone H3 acetylation level in a mouse model of TSC, suggesting that gene expression may be an important factor in TSC pathology [40]. This also raises the possibility of wide alterations of the expression profile in TSC, including for genes encoding synaptic proteins.

Along with the dysfunction of synaptic proteins, dramatic changes in the ultrastructure of the synapses were identified. Since we observed significant dysregulation of specific synaptic proteins in both the cerebral cortex and hippocampus, but statistically significant differences in the number of synaptic vesicles exclusively in the CA2/CA3 region of the hippocampus, it is possible that other proteins not investigated here could be responsible for the observed synaptic overload in the hippocampus. Therefore, the molecular mechanisms responsible for overloaded synaptic hippocampal endings remain unclear and further research is needed to fully understand the link between the activation of mTOR signalling and synapse deregulation.

The alterations of synaptic density in Tsc2^+/−^ mice may be directly related to fluctuations in synaptic proteins. The blurred structure of the synaptic cleft without clearly marked pre- and postsynaptic membranes and a disturbed synaptic membrane were previously observed in experimental models of ASD, in adolescent rat offspring after prenatal exposure to LPS or VPA [33,41]. The synaptic imbalance in TSC was recently suggested to be the result of astrocytes’ impairment [42]. Dooves and co-workers suggested that factors released by affected astrocytes alter the synaptic balance. The presence of giant synapses in the hippocampi of Tsc2^+/−^ animals may also be related to impaired synthesis of synaptic proteins. Interestingly, the impairment of the endoplasmic reticulum, an increased fraction of free polyribosomes, and the impairment of the Golgi apparatus may indicate that local protein synthesis, modification, and transport in Tsc2^+/−-^ animals may be the crucial pathological event triggering further perturbations. Abnormal translation at the synapse was suggested to contribute to the pathological mechanisms of ASD [43].

The local synthesis of synaptic proteins requires energy delivered by synaptic mitochondria [44,45]. Our electron microscopic analysis of mitochondria revealed morphological alterations in Tsc2^+/−^ mice, including common swelling and occasionally excessive elongation. Mitochondrial and bioenergetic anomalies have been observed in patients with ASD and in TSC models [46,47]. Along with these pathological changes in the ultrastructure, our TEM studies indicated the delamination of the myelin sheath in Tsc2^+/–^ animals. This abnormal myelination could contribute to disturbances in processing speed and in neuronal signal transduction. In a recent study by Mühlebner and co-workers, myelin pathology in cortical tubers resected from TSC patients was demonstrated [48]. The level of myelin-associated oligodendrocyte basic protein was decreased and was associated with the presence of autism-related symptoms. Overactivation of the mTOR pathway is probably responsible for the TSC-related impairment of the maturation of oligodendroglia and production of the myelin sheath [49].

In addition, the present results indicate that defects in the mTOR signalling pathway lead to the abnormal enlargement of the brain, megalencephaly. The data are in line with the theory that the dysregulation of mTOR is central to multiple forms of developmental megalencephaly and macrocephaly [50]. The presence of brain enlargement is consistent with the increased brain weight that has also been observed by others [51]. Numerous studies show that mutations in different genes acting as regulators of mTOR signalling and, consequently, the activation of the mTOR pathway lead to megalencephaly or hemimegalencephaly [52,53,54,55,56]. Our results are also in line with previous studies that used a mouse model of TSC, Tsc2^flox/ko^;hGFAP-Cre. In this model, the *Tsc2* gene has been removed from most neurons and glia of the cortex and hippocampus by targeted Cre-mediated deletion in radial glial neuroprogenitor cells. The Tsc2^flox/ko^;hGFAP-Cre mice demonstrated significant brain pathology including brain overgrowth, postnatal megalencephaly, cortical and hippocampal lamination defects, enlarged dysplastic neurons and glia, and astrogliosis [57,58]. Likewise, other studies showed that Tsc1^GFAP^ KO mice developed dramatic, diffuse megalencephaly and higher brain weights compared to control mice [59]. It should also be emphasised that hemimegalencephaly has been demonstrated in some patients with TSC along with abnormal myelination patterns and gyral abnormalities in the enlarged hemisphere [53,60,61,62]. Although megalencephaly and hemimegalencephaly are not typical symptoms of TSC, the case reports confirm the involvement of the activation of the mTOR pathway in processes leading to brain overgrowth, similar to our studies with Tsc2^+/−^ mice. In addition, macrocephaly was observed in children with TSC, but to a lesser extent than in autistic patients [63]. In addition, it was suggested that the overactivation of the mTOR pathway that regulates cell growth, survival, and differentiation could contribute to neuronal hypertrophy and hyperconnectivity leading to network dysfunction and megalencephaly, which, in turn, contributes to the development of autism symptoms [64]. Macrocephaly is present in ca. 15% of patients with idiopathic autism, and head and brain growth is disproportionate to body growth in early childhood [65,66,67]. Therefore, our TSC model effectively recapitulates the brain structure pathology observed in ASD patients with higher mTOR signalling and suggests that disturbances in the mTOR pathway might be responsible for this phenomenon in some autistic patients.

As reported previously, the vast majority of TSC patients have neurological and mental symptoms, including autism, epilepsy, mental retardation, cognitive impairment, attention-deficit hyperactivity disorder, sleep disruption, and anxiety [1]. Recent studies demonstrated that the prevalence of autism in patients with TSC is over 40–50% [68,69,70,71]. The inhibition of mTOR was shown to be an effective strategy for preventing epileptic seizures and behavioural alterations [72]. Treatment with mTOR inhibitors was also demonstrated to be effective in experimental models of autism [73]. Moreover, approximately 50% of TSC patients are developmentally delayed or have an intellectual disability [74].

Experimental animal models successfully replicate only selected typical TSC-related changes. In Tsc2^+/−^ rats, impairment of synaptic plasticity, enhanced episodic-like memory, reduced exploration behaviour, and no differences in anxiety-related behaviour and hippocampus-dependent learning were observed [32,75,76,77]. Additionally, autism-like social deficits were present, and the exploration of novel objects was reduced [76,77]. In Tsc2^+/−^ mice, synaptic plasticity was also affected [78]. Correspondingly, cognitive deficits were observed [79]. Our behavioural analysis demonstrated that, despite the significant impairment of synaptic structure, the animal’s behaviour was almost intact. The exploratory activity, as determined using the open field test, was not changed in Tsc2^+/−^ mice. Additionally, we analysed the numbers of climbings and rearings, and the grooming behaviour, which may indicate changes in anxiety levels in animals. We observed a weak but persistent drift toward increased anxiety in Tsc2^+/−^ mice; however, the difference was without statistical significance. Possibly, the strength of anxiety-like behaviours is very low in Tsc2^+/−^ mice, therefore, much more numerous experimental groups should be tested to confirm this phenomenon.

Because TSC haploinsufficiency is related to autistic behaviours in a high fraction of patients, we analysed the sociability of Tsc2^+/−^ mice in a three-chamber test. The impairment of social behaviour was demonstrated in several experimental models of TSC [27,76,80]. However, in our analysis, Tsc2^+/+^ and Tsc2^+/−^ mice presented the same levels of sociability which is consistent with previously published results of the analysis of the same mouse strain [79,81]. The origin of this inconsistency in experimental models is elusive. It may be caused by the variability in experimental conditions. Another question is the discrepancy between the high frequency of autistic symptoms in TSC patients and the absence of autistic symptoms in our experimental model. We suppose that additional environmental factors may be necessary to induce social behaviour impairment in patients (and animals) with mutations in the *TSC1/2* genes. For example, prenatal viral infection, which is a risk factor for neuropsychiatric disorders, could be such an interfering factor. The data indicate that maternal immune activation (poly(I:C) model) and Tsc2 haploinsufficiency cooperate to disrupt adult social behaviour in mice [81]. Another important factor interacting with TSC1/2 haploinsufficiency could be age. The dynamics of the development of autistic behaviours are not fully understood. It was demonstrated that autistic behaviours may be acquired with age [82].

The most significant behavioural alteration we observed was in the novel object recognition test, which tests hippocampus-dependent memory function [83]. This result may indicate that the learning and memory processes in Tsc2-haploinsufficient mice are slightly impaired compared to those in wild-type mice. However, the result of this test may also have been caused by motor stereotypies. It was recently suggested that the novel object exploration test (not to be confused with the novel object recognition test) may be a potential assay for higher-order repetitive behaviours in mice [84,85]. Repetitive behaviours are a part of autism but may also occur in other conditions such as attention deficit hyperactivity disorder, obsessive-compulsive disorder, and schizophrenia, or even during typical development. Additional studies would be necessary to fully understand the meaning of the reduced ID in the novel object recognition test in Tsc2^+/−^ mice.

Our study supports and extends evidence for the synaptic alterations in TSC. We demonstrate in a rodent model of TSC that inactivation of *Tsc2* gene triggers pathological alterations in synaptic ultrastructure as well as brain overgrowth. In parallel, we indicate changes in the expression of several synaptic proteins that could be responsible for the impairment of synaptic plasticity and memory function.

## 4. Materials and Methods

### 4.1. Chemicals

TRI Reagent, DNase I, and bovine serum albumin (BSA) were purchased from Sigma-Aldrich (St. Louis, MO, USA). Rabbit monoclonal anti-Tsc2 antibody was purchased from Novus Biologicals (Centennial, CO, USA). Mouse monoclonal anti-VAMP1/2 antibody, rabbit polyclonal anti-phospho-synapsin (Ser62/67) antibody, and mouse monoclonal anti-syntaxin-1 were obtained from Santa Cruz Biotechnology Inc. (Santa Cruz, CA, USA). Rabbit polyclonal anti-synaptotagmin-1 antibody and rabbit monoclonal anti-SNAP25 antibody were obtained from Cell Signalling (Beverly, MA, USA). Rabbit polyclonal anti-GAPDH antibody and goat anti-rabbit IgG antibody were purchased from Sigma-Aldrich (St. Louis, MO, USA). Sheep anti-mouse IgG antibody was purchased from GE Healthcare (Little Chalfont, Buckinghamshire, UK). The chemiluminescent reagent Clarity Western ECL Substrate was purchased from Bio-Rad Laboratories (Hercules, CA, USA). The cOmplete protease inhibitor cocktail was purchased from Roche Diagnostics GmbH (Mannheim, Germany). Reagents for reverse transcription (High Capacity cDNA Reverse Transcription Kit) and PCR (TaqMan Fast Advanced Master Mix) were obtained from Thermo Fisher Scientific (Paisley, UK). DMSO and all the other common reagents were from Sigma-Aldrich (St. Louis, MO, USA).

### 4.2. Animals

Young male B6;129S4-Tsc2^tm1Djk/J^ mice (*Mus musculus*) were provided by The Jackson Laboratory and bred in The Laboratory For Genetically Modified Animals of the Mossakowski Medical Research Institute PAS (Warsaw, Poland), which breeds small rodents according to the SPF standard. Animals were analysed at postnatal days 50–60 (PND50–60). The animals were maintained under controlled temperature and humidity conditions with a 12 h light/dark cycle. All of the experiments conducted on animals were approved by the Local Ethics Committee for Animal Experimentation in Warsaw (reference numbers WAW2/081/2019, WAW2/142/2019, and WAW2/045/2020) and were carried out following the EU Directive 2010/63/EU for animal experiments. Every effort was made to minimise the number of animals used and reduce the amount of pain and distress. In the whole study, a group of 60 animals was tested. To avoid the litter effect [86], for every test/assay, data from at least three different litters were analysed.

To determine the genotype, DNA was isolated from the tail as previously described [87]. The PCR was performed in accordance with the protocol recommended by the Jackson Laboratory (https://www.jax.org/strain/004686; accessed on 16 October 2019) in the presence of EvaGreen dye (Biotium, Inc., Fremont, CA, USA). The melt curve analysis was performed using the ABI PRISM 7500 apparatus to distinguish between the different genotypes.

### 4.3. Behavioural Analysis

One week before the first behavioural test, animals were transported to the Behavioural facility anteroom with an inverted day–night phase. Starting from postnatal day (PND) 43, all the animals were subjected, every day, to handling procedures and to the sound-isolated analysis room (overhead diffused light, ca. 12 lux). The behavioural tests were performed by a blinded operator who was not present in the analysis room during the test. The animals’ behaviour was recorded using a Basler acA1300-60 GigE camera (Bassler AG, Germany), and the behaviour was analysed by the blinded operator or calculated using Ethovision XT 10 (Noldus Information Technology, Wageningen, The Netherlands). All the tests were performed in the morning (the beginning of the night phase).

#### 4.3.1. Open-Field Test

The open-field test was performed as previously described [33]. In this test, general locomotor activity, novel environment exploration, and anxiety-related behaviour may be measured. Sixty animals (30 in each experimental group) at 50–51 PND were individually placed in the corner of an open-field chamber (dark grey PCV box, 55 × 55 × 30 cm), and the total distance and the exploration of the central zone (diameter, 36.5 cm) and the border zone were analysed. The numbers of episodes of grooming, rearings, and climbings were counted by the blinded operator.

#### 4.3.2. Novel Object Recognition Test

The test was performed on twenty mice (10 in each group) at PND 54–56, as previously described with modifications [88]. This test exploits the natural tendency of rodents to explore novel objects, for testing non-spatial memory. A day before testing, mice were individually subjected to a habituation session; they were allowed to freely explore the test chamber (a dark grey PCV box, 30 cm × 20 cm × 30 cm) for 5 min. The experimental session consisted of two trials. In the first trial (T1), two identical objects (O1) were placed in the chamber. During the second trial (T2), one object, O1, was replaced with the alternative object, O2. As testing objects, we used a set of plastic bricks and a cell-culture bottle. The objects presented during the sessions were free of olfactory traits, and their positions in the chamber were randomised to eliminate spatial bias in the task. At the beginning of each trial, mice were placed at the centre of the box, with their heads oriented in the opposite direction to the object. The respective durations of T1 and T2 were 5 and 2 min. T2 started 2 h after T1. The basic measurement was the total time spent by mice exploring objects during the T1 and T2 trials. The exploration of an object was defined as follows: directing the nose at a distance of 2 cm to the object and/or touching it with the nose. The climbing time was excluded from the analysis. The index of discrimination (ID) was calculated for each animal and expressed as a ratio: the time spent exploring the novel object/the added time exploring novel and known objects.

#### 4.3.3. Three-Chamber Social Interaction Test (Crawley’s Sociability and Preference for Social Novelty Test)

The three-chamber social interaction test was performed on forty mice (20 in each experimental group), as described previously with modifications [33]. This test assesses general sociability in rodents. The mouse normally prefers to spend more time with another mouse. At PND 58–59, mice were introduced to a three-chamber social interaction apparatus (100 × 55 × 30 cm). Openings between the compartments (10 cm-wide doors) allowed the animals to access all three chambers. In phase I, each tested animal was allowed to explore the environment freely for 10 min for habituation. After the habituation phase (phase I), the subject was gently guided by the operator to the central chamber, and the two entrances were blocked. Two metal wire cages, the first one containing a sex-, age-, and weight-matched mouse and the second one empty, were placed in the left and right chambers (the order was randomised). Then, the two entrances were opened to allow the tested animal to explore the new environment freely for 10 min, and the social preference was measured (social stimulus vs. non-social stimulus)—phase II. Individual movement tracks were recorded by using a video system and analysed using the BehaView 0.0.16 software http://www.pmbogusz.net/?a=behaview (accessed on 24 August 2021). The time spent in each chamber and the time spent on direct interaction with the animal were measured by a blinded operator.

### 4.4. Transmission Electron Microscopy (TEM) Analysis

TEM analysis was performed as described previously [41]. Ten animals (5 in each experimental group) at PND 60 were anaesthetised with ketamine and xylazine (100 and 10 mg/kg, respectively, i.p.) and perfused through the ascending aorta initially with 0.9% NaCl in 0.01 M sodium-potassium phosphate buffer, pH 7.4, and afterward with 2% paraformaldehyde and 2.5% glutaraldehyde in 0.1 M cacodylate buffer, pH 7.4, at 4 °C. Small (ca. 1–2 mm^3^) pieces of brain tissue were dissected: (i) from the brain cortex—primary somatosensory area, layer 1–4; (ii) from the hippocampus—cornu ammonis (CA) subregions CA1/CA2 and subregions CA2/CA3 (both pyramidal layer and stratum radiatum were collected); (iii) from the cerebellum—Crus2 (both molecular and granular layers). All the structures were identified according to the Allen Brain Atlas for the adult mouse (https://atlas.brain-map.org/; accessed on 24 August 2021) [89].

Tissue specimens were post-fixed in the ice-cold fixative solution for 24 h and placed in a mixture of 1% OsO_4_ and 0.8% K_4_[Fe(CN)_6_]. After dehydration in a series of ethanol gradients, the tissue specimens were embedded in epoxy resin (Epon 812) at 60 °C for 24 h. Ultra-thin sections (60 nm) from the somatosensory cortex were stained with uranyl acetate and lead citrate and then examined by transmission electron microscopy (JEM 1011, Jeol, Japan) using a MORADA camera and iTEM 1233 software, operated at 80 kV.

To assess the numbers of synaptic vesicles in nerve terminals, electronograms under a magnification of 50,000 × were taken. The numbers of vesicles in 10 random nerve endings in each animal were counted; in total, 50 nerve endings from 5 animals were analysed. The data were not normally distributed and, therefore, are presented as medians with interquartile ranges, minimums, and maximums.

### 4.5. Analysis of the mRNA Level

The tissue samples for mRNA analysis were collected at PND 60 and stored at −80 °C until analysis. Total RNA was isolated using TRI Reagent (Sigma Aldrich, St. Louis, MO, USA) according to the manufacturer’s protocol, and the digestion of contaminating DNA was performed using DNase I according to the manufacturer’s protocol (Sigma Aldrich, St. Louis, MO, USA). The RNA quantity and quality were checked by spectrophotometric analysis. A reverse transcription was performed using the High Capacity cDNA Reverse Transcription Kit according to the manufacturer’s protocol (Thermo Fisher Scientific, Paisley, UK). Quantitative PCR was performed on an ABI PRISM 7500 apparatus using commercially available TaqMan Gene Expression Assays: Mm01185107_g1 (*Vamp1*), Mm01325243_m1 (*Vamp2*), Mm00436858_m1 (*Syt1*), Mm00444008_m1 (*Stx1a*), Mm02394624_s1 (*Stx1b*), Mm01276449_m1 (*Snap25*), Mm00449772_m1 (*Syn1*), Mm02344307_m1 (*Nlgn1*), Mm01225951_m1 (*Nlgn3*), and Mm00607939_s1 (*Actb*). The relative levels of mRNA were calculated using the ΔΔCt method.

### 4.6. Western Blotting

The tissue samples for WB were collected at PND 60 and stored at −80 °C until analysis. The tissue samples were homogenised, mixed with Laemmli buffer, and denatured at 95 °C for 5 min. After SDS–PAGE, the proteins were transferred to a nitrocellulose membrane under standard conditions and used for immunochemical analysis, followed by chemiluminescent detection. The glyceraldehyde 3-phosphate dehydrogenase (GAPDH) level was analysed as a loading control. In all the experiments, densitometric analysis of the immunoblots was performed using normalization to the immunoreactivity for GAPDH. Densitometric analysis and size-marker-based verification were performed with the TotalLab4 software (NonLinear Dynamics Ltd., Newcastle upon Tyne, UK).

### 4.7. Statistical Analysis

The results are expressed as mean values ± SEMs. In all the analyses, each datapoint is from a separate animal. The normality and equality of the group variances were tested using Shapiro–Wilk tests. Differences between means were analysed using (i) unpaired Student’s *t*-tests or two-way ANOVAs with Bonferroni post-hoc tests for data with normal distributions, or (ii) Mann–Whitney tests for data with non-normal distributions. The level of statistical significance was set at *p* < 0.05. The statistical analyses were performed using Graph Pad Prism version 8.0 (Graph Pad Software, San Diego, CA, USA).

## Figures and Tables

**Table 1:** The effect of Tsc2 haploinsufficiency on the mRNA levels for synaptic proteins.

	Hippocampus		Cortex		Cerebellum	
mRNA	Tsc2^+/+^	Tsc2^+/−^	Tsc2^+/+^	Tsc2^+/−^	Tsc2^+/+^	Tsc2^+/−^
*Vamp1*	1.00 ± 0.05	0.99 ± 0.06	1.00 ± 0.05	1.15 ± 0.03 *	1.00 ± 0.07	0.94 ± 0.08
*Vamp2*	1.00 ± 0.04	0.96 ± 0.03	1.00 ± 0.03	1.22 ± 0.04 **	1.00 ± 0.03	0.95 ± 0.08
*Syt1*	1.00 ± 0.03	0.96 ± 0.04	1.00 ± 0.03	1.13 ± 0.04 *	1.00 ± 0.05	0.99 ± 0.08
*Stx1a*	1.00 ± 0.07	0.78 ± 0.04	1.00 ± 0.05	1.11 ± 0.04	1.00 ± 0.24	0.87 ± 0.06
*Stx1b*	1.00 ± 0.04	0.94 ± 0.02	1.00 ± 0.02	1.00 ± 0.02	1.00 ± 0.03	0.88 ± 0.06
*Snap25*	1.00 ± 0.08	0.95 ± 0.04	1.00 ± 0.05	1.13 ± 0.03	1.00 ± 0.04	0.84 ± 0.09
*Syn1*	1.00 ± 0.04	1.11 ± 0.02	1.00 ± 0.06	1.07 ± 0.07	1.00 ± 0.08	1.00 ± 0.07
*Nlgn1*	1.00 ± 0.09	0.95 ± 0.08	1.00 ± 0.03	1.30 ± 0.03 ***	1.00 ± 0.09	1.04 ± 0.07
*Nlgn3*	1.00 ± 0.05	1.06 ± 0.02	1.00 ± 0.05	0.98 ± 0.02	1.00 ± 0.02	0.96 ± 0.04

The levels of mRNA in the hippocampus, cortex, and cerebellum were determined by using real-time PCR and calculated using the ΔΔCt method, with *Actb* as a reference gene. Data presented in the table are relative quantity (RQ) ± SEM (*n* = 3–6). Each sample was from a separate animal. Samples were isolated from at least three different litters. * *p* < 0.05, ** *p* < 0.01, and *** *p* < 0.01, compared with the control group, as determined using Student’s *t*-test.

**Table 2:** The effect of Tsc2 haploinsufficiency on levels of synaptic proteins—densitometric analysis.

	Hippocampus		Cortex		Cerebellum	
Protein	Tsc2^+/+^	Tsc2^+/−^	Tsc2^+/+^	Tsc2^+/−^	Tsc2^+/+^	Tsc2^+/−^
Vamp1/2	1.00 ± 0.14	1.56 ± 0.08 *	1.00 ± 0.08	1.46 ± 0.19 *	1.00 ± 0.09	1.59 ± 0.17 *
Syt1	1.00 ± 0.09	1.04 ± 0.05	1.00 ± 0.04	0.86 ± 0.04 *	1.00 ± 0.21	1.03 ± 0.19
Stx1	1.00 ± 0.10	0.95 ± 0.12	1.00 ± 0.05	0.80 ± 0.03 **	1.00 ± 0.13	1.10 ± 0.14
Snap25	1.00 ± 0.19	0.82 ± 0.09	1.00 ± 0.18	0.73 ± 0.08	1.00 ± 0.30	1.07 ± 0.33
p-Syn	1.00 ± 0.11	1.77 ± 0.11 **	1.00 ± 0.23	1.94 ± 0.24 *	1.00 ± 0.11	1.20 ± 0.18
Nlgn1	1.00 ± 0.08	1.09 ± 0.02	1.00 ± 0.07	1.08 ± 0.15	not detected	not detected
Nlgn3	1.00 ± 0.07	1.13 ± 0.08	1.00 ± 0.11	1.15 ± 0.23	not detected	not detected

The immunoreactivity of synaptic proteins in the hippocampus, cortex, and cerebellum was determined by using Western blotting. GAPDH was used as a loading control. Results of the densitometric analysis were normalised to GAPDH and calculated as relative changes and are presented as the mean value ± SEM. (*n* = 3–6). Data were analysed using Student’s *t*-test. * *p* < 0.05 and ** *p* < 0.01. Each sample was from a separate animal. Samples were isolated from at least three different litters.

**Table 3:** Pathological alterations of neuronal ultrastructure found in the brains of Tsc2^+/−^ mice.

	CA1/CA2	CA2/CA3	Cortex	Cerebellum
blurred structure of synaptic density	+	+	+	+
overloaded synaptic endings	+	+	+/–	+/–
swollen mitochondria	+	+	+	+
excessively elongated mitochondria	+	+	+	+
polyribosomes in the cytoplasm	+	+	+	+
swollen Golgi apparatus	+	+	+	+
vacuolar structures in the cell nucleus	+	+	+	+
delamination of myelin	+	+	+	+

+ alteration frequently observed in the tissue of Tsc2^+/−^ animals; +/– alteration exceptionally observed in the tissue of Tsc2^+/−^ animals.

## Data Availability

The raw data supporting the conclusions of this article will be made available by the authors, without undue reservation.
